# Use of the Consumer-Based Meditation App Calm for Sleep Disturbances: Cross-Sectional Survey Study

**DOI:** 10.2196/19508

**Published:** 2020-11-13

**Authors:** Jennifer Huberty, Megan E Puzia, Linda Larkey, Michael R Irwin, Ana-Maria Vranceanu

**Affiliations:** 1 College of Health Solutions Arizona State University Phoenix, AZ United States; 2 Behavioral Research and Analytics, LLC Salt Lake City, UT United States; 3 Edson College of Nursing and Health Innovation Arizona State University Phoenix, AZ United States; 4 Cousins Center for Psychoneuroimmunology University of Calfornia, Los Angeles Los Angeles, CA United States; 5 Mindful Awareness Research Center University of Calfornia, Los Angeles Los Angeles, CA United States; 6 Jane and Terry Semel Insitute for Neuroscience and Human Behavior University of Calfornia, Los Angeles Los Angeles, CA United States; 7 Department of Psychiatry and Biobehavioral Sciences David Geffen School of Medicine University of Calfornia, Los Angeles Los Angeles, CA United States; 8 Integrated Brain Health Clinical and Research Program Department of Psychiatry Massachusetts General Hospital/Harvard Medical School Cambridge, MA United States

**Keywords:** insomnia, mental health, mindfulness, meditation, mobile apps, consumer behavior, mobile phone

## Abstract

**Background:**

Over 30% of Americans report regular sleep disturbance, and consumers are increasingly seeking strategies to improve sleep. Self-guided mindfulness mobile apps may help individuals improve their sleep. Despite the recent proliferation of sleep content within commercially available mindfulness apps, there is little research on how consumers are using these apps for sleep.

**Objective:**

We conducted a cross-sectional survey among subscribers to Calm, a popular, consumer-based, mindfulness-based meditation app, and described and compared how good sleepers, poor sleepers, and those with self-reported insomnia use the app for sleep.

**Methods:**

Participants who were paying subscribers of Calm and had used a sleep component of Calm in the last 90 days were invited to complete an investigator-developed survey that included questions about sleep disturbance and the use of Calm for sleep. Based on self-reports of sleep disturbances and of insomnia diagnosis, participants were categorized as “good sleepers,” “poor sleepers,” or “those with insomnia diagnosis.” Chi-square tests compared reasons for downloading the app and usage patterns across participants with and without sleep disturbance.

**Results:**

There was a total of 9868 survey respondents. Approximately 10% of participants (1008/9868, 10.21%) were good sleepers, 78% were poor sleepers (7565/9868, 77.66%), and 11% reported a diagnosis of insomnia (1039/9868, 10.53%). The sample was mostly White (8185/9797, 83.55%), non-Hispanic (8929/9423, 94.76%), and female (8166/9578, 85.26%). The most common reasons for sleep disturbances were racing thoughts (7084/8604, 82.33%), followed by stress or anxiety (6307/8604, 73.30%). Poor sleepers and those with insomnia were more likely than good sleepers to have downloaded Calm to improve sleep (χ^2^_2_=1548.8, *P*<.001), reduce depression or anxiety (χ^2^_2_=15.5, *P*<.001), or improve overall health (χ^2^_2_=57.6, *P*<.001). Respondents with insomnia used Calm most often (mean 5.417 days/week, SD 1.936), followed by poor sleepers (mean 5.043 days/week, SD 2.027; *F*_2_=21.544, *P*<.001). The most common time to use Calm was while lying down to sleep (7607/9686, 78.54%), and bedtime use was more common among poor sleepers and those with insomnia (χ^2^_2_=382.7, *P*<.001). Compared to good and poor sleepers, those with insomnia were more likely to use Calm after waking up at night (χ^2^_2_=410.3, *P*<.001). Most participants tried to use Calm on a regular basis (5031/8597, 58.52%), but regular nighttime use was most common among those with insomnia (646/977, 66.1%), followed by poor sleepers (4040/6930, 58.30%; χ^2^_2_=109.3, *P*<.001).

**Conclusions:**

Of the paying subscribers to Calm who have used one of the sleep components, approximately 90% have sleep difficulties, and 77% started using Calm primarily for sleep. These descriptive data point to areas of focus for continued refinement of app features and content, followed by prospective trials testing efficacy of consumer-based meditation mobile apps for improving sleep.

## Introduction

A large body of research suggests that mindfulness has positive effects on both mental and physical health [1-3]. In recent years, there has been a noticeable surge in the use of commercially available mindfulness-based smartphone apps [4,5], and there is growing evidence that individuals are using these apps specifically to help improve their health, mood, and sleep [6]. Because content is consistently available and accessible to users whenever it is needed, mindfulness apps provide users with just-in-time resources to navigate feelings of anxiety, stress, or frustration [7], thus reinforcing resilient responses to stressors [7]. Hence, mindfulness apps represent a convenient, cost-effective way to improve health and well-being [6,8].

Sleep disturbance is prevalent in over 30% of the general population, with rates increasing over the last decade and contributing to mental and physical health problems [9,10]. Consumers are increasingly seeking strategies to improve sleep. Almost 75% of US adults own smartphones, and apps for sleep have become highly popular [11]. However, most apps that are specifically designed for sleep are used to measure or observe sleep, rather than deliver an intervention to improve sleep [11]. Although recent randomized controlled trials (RCTs) provide preliminary evidence supporting the benefits of mindfulness meditation for improving sleep quality [3], reviews of sleep app content and delivery indicate that most apps focus on traditional cognitive behavioral interventions and/or provision of sleep education and none are based on mindfulness meditation strategies [11,12].

Given the growing evidence of the value of meditation for improving sleep, and the increase in popular knowledge about this benefit, it is likely that sleep concerns are becoming a driving force in consumers’ selection of meditation apps. In a recent survey of 12,151 subscribers to one of the most widely disseminated meditation apps, Calm, over 75% reported having some form of sleep disturbance at the time of purchase, and improving sleep was one of the primary reasons for downloading the app [13]. Moreover, those who reported sleep difficulties were more likely to use Calm on a regular basis.

To address users’ sleep-specific concerns, developers of commercial mindfulness apps have begun to diversify content and add components designed to target sleep [14]. For example, in addition to their library of mindfulness meditations, Calm has developed narrated sleep programs that use traditional literary and storytelling methods that listeners can use to help fall asleep (ie, Sleep Stories), meditations that are specifically created to improve sleep (eg, to help listeners fall asleep at night and to help develop a positive mindset surrounding sleep), and a curated collection of relaxing music and soundscapes that are softer, have a slower tempo, and are generally longer in duration (eg, 30-60 minutes) than some of the other music and soundscape tracks available in the app. Other meditation apps, such as Headspace and Insight Timer, have also begun to incorporate sleep-specific content. Despite the recent proliferation of sleep content within commercially available mindfulness apps, there is little research related to the characteristics of individuals who use these apps for sleep and/or how they are using apps for sleep [15]. For example, we do not currently know much about the demographics of users, how they use sleep content, or the specific reasons that they use these apps for sleep (eg, to fall asleep, to prevent early waking, to get restful sleep). A more nuanced understanding of consumers’ sleep-related usage patterns is needed in order to personalize these strategies within mindfulness apps, which has the potential to augment adherence to these apps and enhance their efficacy in improving sleep disturbance.

To address this knowledge gap, we conducted a cross-sectional survey among subscribers to the Calm mobile app who had accessed at least one sleep-related feature in the past 90 days. First, we categorized participants as good sleepers, poor sleepers, or those diagnosed with insomnia, and described the types of specific sleep disturbance reported within each category (eg, falling asleep, staying asleep, not waking too early, and getting restful sleep). Next, we described and compared the self-reported app engagement of good sleepers, poor sleepers, and those with insomnia, including reasons for downloading the app, frequency of use, and how they use the app for sleep.

## Methods

### Ethics Approval

The Institutional Review Board at Arizona State University approved this study (STUDY00011083). All participants provided electronic consent before participating in the survey. The data sets generated or analyzed during the study are available from the corresponding author upon request.

### Study Design and Recruitment

The study was cross-sectional. Participants were paying subscribers to the Calm meditation app. Participants were recruited via email in December 2019. Calm subscribers were sent a recruitment email if they had (1) completed at least one session of Calm using a sleep-related component in the last 90 days, so as to enrich the sample with those who used sleep-related app content, and (2) opened at least one email from Calm within the last 90 days, to target more active users who would be more likely to see and open the email. Prior to consenting, potential participants were asked to verify their eligibility by confirming that they (1) were at least 18 years old and (2) were able to read and understand questions written in English. Eligible subscribers were then directed to an electronic consent form stating that by choosing to continue to the survey questions on the next page, they were agreeing to participate. They were informed that at the conclusion of the survey, they would have the option to provide their email address to be included in a drawing for one of two US $99 Amazon gift cards.

### Survey

Participants completed a demographic questionnaire followed by an investigator-developed quantitative survey that included seven questions about using Calm for sleep, which are presented in Textbox 1 (see Multimedia Appendix 1 for the complete sleep survey). Specifically, there were three questions about whether or not sleep difficulties were experienced and, if so, the types of sleep disturbance participants were experiencing when they initially downloaded Calm (ie, type of difficulties, reasons for sleep difficulties, and use of sleep aids), and four questions about their app engagement (ie, reason for downloading Calm, frequency and time of Calm use, and how Calm was used at night). The survey included branching logic so that questions were only presented if they were relevant to the participant based on their previous responses describing their sleep disturbance and app engagement (eg, those who reported that they did not have sleep disturbance were not asked about the reasons they had disturbed sleep).

**Survey questions used in the analyses.**

When you downloaded Calm, had you been diagnosed with insomnia? Y/N

When you first started using Calm, did you have difficulty falling asleep or staying asleep? (Check all that apply.)

- Falling asleep
- Staying asleep
- Getting restful sleep
- Waking up too early
- None of the above (ie, no sleep difficulties)

What are some reasons why you think you have trouble sleeping? (Check all that apply.)

- Racing thoughts or not being able to turn your mind off
- Stress or anxiety
- Nightmares
- Noise in the environment
- Light in the environment
- Work or sleep schedules (eg, working late at night and taking late-afternoon naps)
- Fluctuating hormones
- Physical pain or discomfort
- Medications that interfere with sleep
- Caffeine
- Other(s)
- I don’t know

During the last 90 days: Aside from Calm, what other things have you used to help you sleep? (Check all that apply.)

- Professional medical treatment (eg, sleep specialist)
- Professional psychological treatment for sleep
- Prescription sleep medications
- Over-the-counter sleep medications (not melatonin)
- Melatonin
- Noise machine
- ASMR (autonomous sensory meridian response) videos and audio
- Relaxation exercises and routines (eg, progressive muscle relaxation)
- Yoga before bed
- Other relaxation apps
- Other activities

Why did you download Calm? (Check all that apply.)

- Improve overall health
- Reduce stress
- Reduce depression or anxiety
- Improve sleep
- Friend recommended the app
- Someone bought it for me
- Curious
- Other

Generally, when do you use Calm? (Check all that apply.)

- Within the 30 minutes after waking up in the morning
- In the morning, but not within 30 minutes of waking up
- In the afternoon
- In the evening
- At night, but not within 30 minutes of going to bed
- Within the 30 minutes before laying down to go to bed at night
- While laying down to go to bed at night (eg, to fall asleep)
- When I wake up during the night

Which best describes the way you use Calm at night? (Check one.)

- I try to use Calm at night on a regular basis
- I sometimes or occasionally use Calm at night
- I use Calm at night only when I need it (eg, because of sleep difficulties that night)

When you use Calm at night because you need it, what do you usually need it for? (Check all that apply.)

- Falling asleep
- Staying asleep
- Falling back to sleep after I wake up
- Getting restful sleep
- Waking too early

### Defining Good Sleepers, Poor Sleepers, and Those With Insomnia Diagnosis

Respondents were categorized based on their self-reports regarding (1) the presence or absence of sleep disturbance at the time they downloaded Calm and (2) the presence or absence of an insomnia diagnosis at the time they downloaded Calm. *Good sleepers* reported their sleep disturbance to be “none (ie, no sleep difficulties).” *Poor sleepers* reported experiencing at least one type of sleep disturbance (ie, difficulty falling asleep, staying asleep, waking too early, or getting restful sleep) but *did not have* a diagnosis of insomnia. Those who endorsed at least one type of sleep disturbance and self-reported a diagnosis of insomnia were categorized as such (ie, *insomnia diagnosis*).

### Statistical Analysis

Data were analyzed using SPSS Statistics for Mac, version 26.0 (IBM Corp). Chi-square tests were used to compare sleep characteristics and health diagnoses across participants with and without sleep difficulties. Chi-square tests were also used to compare sleep characteristics, reasons for downloading Calm, and app-usage patterns across participants with and without sleep difficulties. Significant chi-square tests were followed up with *z* tests of column proportions using Bonferroni-corrected *P* values to adjust for multiple comparisons.

## Results

### Overview

Of the 366,173 subscribers who received an invitation to participate, 130,849 opened the invitations (35.73%). There were 19,341 subscribers who clicked the link to the survey (14.78% of those who opened the email), 14,642 (11.19%) consented to participate, and 11,095 completed the full survey (completion rate 75.78%). This completion rate was likely negatively impacted by the high volume of responses immediately following the release of survey invitations, which initially overtaxed host servers such that survey loading times were substantially slowed (see Limitations section below).

Although all participants had completed at least one session with a Calm sleep component, participants were included in the analyses only if they reported that they used at least one Calm component for the specific purpose of helping their sleep (9868/11,095, 88.94% of survey completers). Approximately 10% of participants were classified as good sleepers (ie, no sleep disturbance; 1008/9868, 10.21%), 78% were poor sleepers (ie, at least one type of sleep disturbance but without an insomnia diagnosis; 7565/9868, 77.66%), and 11% reported a diagnosis of insomnia (ie, at least one type of sleep disturbance and an insomnia diagnosis; 1039/9868, 10.53%). The remaining participants (185/9868, 1.87% of survey completers) were excluded from the analyses because they either (1) reported an insomnia diagnosis but did not endorse any type of sleep disturbance (27/9868, 0.27% of survey completers) or (2) they did not respond to questions about sleep disturbance or health diagnoses (158/9868, 1.60% of survey completers).

### Demographic Characteristics

On average, respondents were 47.2 years old (SD 14.1). The majority were White, non-Hispanic, and female (see Table 1). The median household income was US $80,000 (IQR US $50,000-$130,000), and most participants had received a higher education degree and were employed.

**Table 1:** Demographic characteristics of the sample (N=9868).

Category		Values, n (%)
**Race (n=9797)**		
	White, European American, or Caucasian	8185 (83.55)
	Asian or Asian American	307 (3.13)
	Black, African American, or Native African	238 (2.43)
	Biracial or multiracial	270 (2.76)
	Other	411 (4.20)
**Ethnicity (n=9423)**		
	Non-Hispanic	8929 (94.76)
	Hispanic	494 (5.24)
**Gender (n=9578)**		
	Female	8166 (85.26)
	Male	1380 (14.41)
	Other	32 (0.33)
**Education (n=9531)**		
	11th grade or less	40 (0.42)
	High school or General Educational Development	454 (4.76)
	Some college	967 (10.15)
	2-year college or technical school degree	936 (9.82)
	Bachelor’s degree	3608 (37.86)
	Graduate degree	3397 (35.64)
	Other	129 (1.35)
**Employment status (n=9500)**		
	Full-time employment	5933 (62.45)
	Part-time employment	1170 (12.32)
	Unemployed	338 (3.56)
	Disability	234 (2.46)
	Full-time student	279 (2.94)
	Other	1546 (16.27)

### Types of Sleep Disturbances

The most common type of sleep disturbance was difficulty falling asleep, which was endorsed by 73% of those reporting a sleep disturbance (5523/7565, 73.00%) (see Table 2).

**Table 2:** Types of sleep disturbance reported by poor sleepers and respondents with insomnia.

Type of sleep disturbance	Poor sleepers (n=7565), n (%)	Those with self-reported insomnia diagnosis (n=1039), n (%)
Falling asleep	5523 (73.01)	848 (81.62)
Staying asleep	4012 (53.03)	724 (69.68)
Getting restful sleep	3488 (46.11)	603 (58.04)
Waking up too early	1182 (15.62)	296 (28.49)

Among those who reported sleep disturbance, the most commonly reported reasons for having sleep disturbance were racing thoughts (6220/7565, 82.22%), followed by stress or anxiety (5508/7565, 72.81%) (see Table 3). Other reasons, such as pain, were relatively infrequent, occurring in less than 20% of poor sleepers or those with insomnia. Attribution of sleep problems to racing thoughts did not differ between poor sleepers and those with insomnia; however, compared to poor sleepers, respondents with insomnia were significantly more likely to report that sleep difficulties were caused by stress or anxiety, physical pain, fluctuating hormones, nightmares, or use of medications that interfere with sleep.

**Table 3:** Reasons for sleep disturbance reported by poor sleepers and respondents with insomnia.

Reason	Poor sleepers (n=7565), n (%)	Those with self-reported insomnia diagnosis (n=1039), n (%)	Chi-square (*df* 1)	*P* value^a^	*φ*
Racing thoughts	6220 (82.22)	864 (83.16)	0.6	.46	.008
Stress or anxiety	5508 (72.81)	799 (76.90)	7.8	.005	.030
Physical pain or discomfort	1437 (19.00)	354 (34.07)	126.0	<.001	.121
Fluctuating hormones	1446 (19.11)	243 (23.39)	10.6	.001	.035
Noise in the environment	1187 (15.69)	176 (16.94)	1.1	.30	.011
Work or sleep schedule	858 (11.34)	128 (12.32)	0.9	.35	.010
Nightmares	512 (6.77)	142 (13.67)	61.9	<.001	.085
Light in the environment	503 (6.65)	85 (8.18)	3.4	.07	.020
Caffeine	569 (7.52)	59 (5.68)	4.6	.03	.023
Medications that interfere with sleep	256 (3.38)	112 (10.78)	122.0	<.001	.119
Others	379 (5.01)	73 (7.03)	7.5	.006	.029
I don’t know	188 (2.49)	29 (2.79)	0.3	.56	.006

^a^Tests are adjusted for multiple comparisons within the table using the Bonferroni correction.

Across all participants, rates of reported use of sleep aids were highest among those with insomnia and lowest among good sleepers, with the exception of practicing yoga before bed, which did not significantly differ across sleeper types (see Table 4). Those with insomnia were the most likely to use almost all listed sleep aids, followed by poor sleepers, and then good sleepers; all pairwise differences were significant. However, good sleepers were more likely than poor sleepers and those with insomnia to report using other relaxation apps for sleep, although poor sleepers and those with insomnia did not differ with regard to the use of other apps for sleep.

**Table 4:** Use of sleep aids reported by good sleepers, poor sleepers, and respondents with insomnia.

Type	Good sleepers (n=1008), n (%)	Poor sleepers (n=7565), n (%)	Those with self-reported insomnia diagnosis (n=1039), n (%)	Chi-square (*df* 2)	*P* value^a^	*φ*
Melatonin	143 (14.19)	2243 (29.65)	429 (41.29)	183.7	<.001	.138
Over-the-counter sleep medications	54 (5.36)	1566 (20.70)	337 (32.44)	233.9	<.001	.156
Prescription sleep medications	47 (4.66)	1077 (14.24)	565 (54.38)	1145.9	<.001	.345
Relaxation exercises and routines	181 (17.96)	2017 (26.66)	377 (36.28)	87.9	<.001	.096
Noise machine	138 (13.69)	1317 (17.41)	239 (23.00)	31.7	<.001	.057
Yoga before bed	146 (14.48)	1149 (15.19)	158 (15.21)	0.4	.84	.006
Professional medical sleep treatment	16 (1.59)	344 (4.55)	209 (20.12)	435.5	<.001	.213
Professional psychological treatment	11 (1.09)	255 (3.37)	162 (15.59)	350.6	<.001	.191
ASMR^b^ videos and audio	28 (2.78)	357 (4.72)	67 (6.45)	15.4	<.001	.040
Other activities	166 (16.47)	1154 (15.25)	124 (11.93)	9.7	.008	.032
Other relaxation apps	74 (7.34)	757 (10.01)	134 (12.90)	17.5	<.001	.043

^a^Tests are adjusted for multiple comparisons within the table using the Bonferroni correction.

^b^ASMR: autonomous sensory meridian response.

### App Engagement by Type of Reported Sleep Disturbance

#### Reasons for Downloading Calm

The reasons for downloading Calm varied in relation to the presence of sleep disturbance. As expected, those with poor sleep and insomnia were more likely to cite improving sleep as their reason for downloading Calm. Poor sleepers and those with insomnia were also more likely to report additional reasons for downloading the app as compared to good sleepers, including to reduce depression and anxiety and to improve overall health (see Table 5).

**Table 5:** Reasons for downloading Calm reported by good sleepers, poor sleepers, and respondents with insomnia.

Reason	Good sleepers (n=1008), n (%)	Poor sleepers (n=7565), n (%)	Those with self-reported insomnia diagnosis (n=1039), n (%)	Chi-square (*df* 2)	*P* value^a^	*φ*
Improve sleep	286 (28.37)	6186 (81.77)	937 (90.18)	1548.8	<.001	.401
Reduce stress	602 (59.72)	4443 (58.73)	572 (55.05)	5.9	.054	.025
Reduce depression and anxiety	477 (47.32)	3398 (44.92)	532 (51.20)	15.5	<.001	.040
Improve overall health	392 (38.89)	2080 (27.50)	283 (27.24)	57.6	<.001	.077
Curious	232 (23.02)	1186 (15.68)	104 (10.01)	65.6	<.001	.083
Friend recommended	146 (14.48)	1070 (14.14)	134 (12.90)	1.4	.51	.012
Gift	25 (2.48)	96 (1.27)	15 (1.44)	9.4	.009	.031
Other	135 (13.39)	392 (5.18)	38 (3.66)	118.8	<.001	.111

^a^Tests are adjusted for multiple comparisons within the table using the Bonferroni correction.

#### Frequency of Using Calm

There were significant differences in how often good sleepers, poor sleepers, and those with insomnia used Calm (*F*_2_=21.544, *P*<.001). Respondents with an insomnia diagnosis used Calm most often, on average 5.417 (SD 1.936) times per week, followed by poor sleepers, who used Calm 5.043 (SD 2.027) times per week, and then good sleepers, who used Calm 4.852 (SD 2.165) times per week. All pairwise comparisons were statistically significant (insomnia > poor sleepers, *P*<.001; insomnia > good sleepers, *P*<.001; poor sleepers > good sleepers, *P*=.02).

#### Using Calm for Sleep

When asked about the most frequent times to use Calm, in general (ie, not specific to using for sleep), all sleeper types reported that the most common time they used Calm was while lying down to go to bed (7607/9686, 78.54%). Poor sleepers and those with insomnia were significantly more likely to use Calm while lying down to go to bed than good sleepers (see Table 6). Poor sleepers were also significantly more likely than good sleepers to report using Calm after waking up at night, but using Calm to fall back asleep was most common among those with insomnia.

Participants significantly differed with regard to how they used Calm at night (^2^_2_=109.3, *P*<.001, *φ*=.107). Among those who used Calm at night (8597/9612, 89.44%), most reported that they tried to use Calm at night on a regular basis (5031/8597, 58.52%). Regular nighttime use was most common among those with insomnia, followed by poor sleepers (see Table 7).

**Table 6:** Time of day to use Calm reported by good sleepers, poor sleepers, and respondents with insomnia.

Time	Good sleepers (n=1008), n (%)	Poor sleepers (n=7565), n (%)	Those with self-reported insomnia diagnosis (n=1039), n (%)	Chi-square (*df* 2)	*P* value^a^	*φ*
Within the 30 minutes after waking up in the morning	257 (25.50)	1112 (14.70)	127 (12.22)	88.8	<.001	.096
In the morning, but not within 30 minutes of waking up	249 (24.70)	916 (12.11)	106 (10.20)	132.2	<.001	.117
In the afternoon	232 (23.02)	1097 (14.50)	156 (15.01)	49.5	<.001	.072
In the evening	229 (22.72)	1427 (18.86)	234 (22.52)	14.4	.001	.039
At night, but not within 30 minutes of going to bed	69 (6.85)	360 (4.76)	61 (5.87)	9.4	.009	.031
Within the 30 minutes before laying down to go to bed at night	226 (22.42)	1531 (20.24)	239 (23.00)	6.1	.047	.025
While laying down to go to bed at night (eg, to fall asleep)	559 (55.46)	6200 (81.96)	848 (81.62)	382.7	<.001	.200
When I wake up during the night and I can’t fall back asleep	185 (18.35)	3759 (49.69)	610 (58.71)	410.3	<.001	.207

^a^Tests are adjusted for multiple comparisons within the table using the Bonferroni correction.

**Table 7:** Routine, occasional, and as-needed nighttime use of Calm reported by good sleepers, poor sleepers, and respondents with insomnia.

Usage pattern	Good sleepers (n=690), n (%)	Poor sleepers (n=6930), n (%)	Those with self-reported insomnia diagnosis (n=977), n (%)
I try to use Calm at night on a regular basis	345 (50.0)	4040 (58.30)	646 (66.1)
I sometimes or occasionally use Calm at night	219 (31.7)	1247 (17.99)	130 (13.3)
I use Calm at night only when I need it	126 (18.3)	1643 (23.71)	201 (20.6)

Among those who used Calm at night only when they needed it, it was used most for falling asleep, followed by falling back to sleep after waking (see Table 8). Reasons for needing Calm also differed across participants. As shown, poor sleepers and those with insomnia were more likely than good sleepers to need Calm to fall asleep. Those with insomnia were the most likely to report needing Calm to fall back to sleep after waking, followed by poor sleepers. There were no differences with regard to needing Calm to stay asleep, get restful sleep, or not wake up too early.

**Table 8:** Reasons for as-needed nighttime use of Calm reported by good sleepers, poor sleepers, and respondents with insomnia.

Reason	Good sleepers (n=126), n (%)	Poor sleepers (n=1643), n (%)	Those with self-reported insomnia diagnosis (n=201), n (%)	Chi-square (*df* 2)	*P* value^a^	*φ*
Falling asleep	95 (75.4)	1270 (77.30)	153 (76.1)	0.4	.84	.013
Falling back to sleep after waking up	39 (31.0)	922 (56.12)	133 (66.2)	40.3	<.001	.143
Getting restful sleep	23 (18.3)	336 (20.45)	41 (20.4)	0.4	.84	.013
Staying asleep	5 (4.0)	343 (20.88)	53 (26.4)	25.6	<.001	.114
Waking up too early	4 (3.2)	144 (8.76)	19 (9.5)	5.0	.08	.050

^a^Tests are adjusted for multiple comparisons within the table using the Bonferroni correction.

## Discussion

### Principal Findings

We conducted a cross-sectional survey of subscribers to the Calm mobile app that had used a sleep-related component within the past 90 days. First, we categorized participants as good sleepers, poor sleepers, or having a diagnosis of insomnia, and described the specific types of sleep disturbance reported by individuals within each category (eg, falling asleep, staying asleep, not waking too early, and getting restful sleep). Next, we described and compared how good sleepers, poor sleepers, and those with insomnia engage with Calm, including reasons for downloading the app, frequency of app usage, and how the app was used specifically for sleep. This is the first study to report the use of mindfulness meditation apps for sleep among mobile app consumers.

Only 10% of the respondents indicated they had no difficulty with sleep. Given the high rates of sleep disturbance among US adults, it is not surprising that sleep disturbance is highly represented in our sample. We do, however, acknowledge that the rates of sleep disturbance and insomnia in our sample may be inflated, due to our specific recruitment of subscribers who had recently used sleep components of the Calm app. Despite this, it is of interest to describe this sample and examine their perceptions regarding the causes of their sleep disturbance, other sleep remedies they may be exploring, and how they use Calm for sleep.

Across the entire sample, the most common type of sleep disturbance reported was difficulty falling asleep, closely followed by difficulty staying asleep. Poor sleepers and those with insomnia indicated that the most common reasons for disturbed sleep were racing thoughts and stress or anxiety; however, reports of sleep disturbance due to stress or anxiety were more prevalent among those with insomnia than in poor sleepers without insomnia. These results are consistent with both the larger sleep-related literature [9,10,16] and insomnia-specific literature [17-19]. Further, findings are consistent with research on the comorbidity between sleep and anxiety disorders [9,10,16].

Those with insomnia were more likely than good or poor sleepers to use Calm to help them sleep, and they used Calm for sleep more often. Those with insomnia diagnoses were also more likely to use Calm in conjunction with other sleep aids. This is consistent with research showing that current treatment options for insomnia and sleep disturbance (eg, pharmacological and behavioral therapies) are not meeting the needs of individuals in this population [20]. Given this, mindfulness-based apps that can deliver sleep-specific tools, on demand, may be an innovative strategy to improve sleep difficulties and self-manage sleep, beyond other treatment options [21].

Although most of Calm’s sleep content is designed for general relaxation and is intended to allow for flexible usage at any time of day, some components (eg, Sleep Stories) are specifically designed to be used right before bed, such that it is not surprising that most participants used Calm at night. However, rates of nighttime usage were significantly higher among poor sleepers and those with insomnia compared to rates among good sleepers (eg, 81% vs 51% used at bedtime). Although both poor sleepers and those with insomnia primarily used Calm when laying down for bed to fall asleep, those with insomnia were significantly more likely than poor sleepers to use Calm to fall *back* asleep after waking up at night. While falling asleep may be primarily a function of high presleep arousal, insomnia is often characterized by middle-of-the-night awakenings and challenges falling back asleep [22,23]. Problems with middle-of-the-night awakening may, to some extent, differentiate less severe sleep disturbance from more severe sleep disturbance that may warrant diagnosis and clinical attention [22,23], highlighting a specific need within this population. Our findings show that, in addition to using Calm to fall asleep at night, some Calm subscribers with insomnia may also be using the app as a means of returning to sleep after nighttime awakenings.

Additional research is needed to assess the app content that is most attractive to, and utilized by, Calm users with sleep disturbance, including those with insomnia diagnoses, and to test the effects of different types of sleep content on sleep improvements. Findings from this line of research will likely be relevant in determining the extent to which specific results may generalize to other mindfulness meditation apps. Additionally, of course, these same questions should be pursued in examining sleep-related and general meditation features of other mindfulness-based apps.

When asked to describe how they used Calm at night, most participants reported that they “try to use Calm at night on a regular basis” (ie, rather than using it on an occasional or as-needed basis). This finding is encouraging and suggests perceived benefit in using the app for regular sleep. Interestingly, good sleepers were almost twice as likely to report that they used Calm at night sometimes or occasionally, as opposed to most nights. Research on adherence to sleep interventions indicates that participants who perceive the intervention to be relevant or necessary are more consistently engaged in their prescribed treatment [24,25]. Future studies using app-based interventions for sleep should consider insomnia, or severity of baseline sleep disturbance, as a potential moderator of adherence and, by extension, treatment effects. Additionally, given that some poor sleepers and participants with insomnia (over 20%) reported that they use Calm for sleep only when experiencing acute or immediate sleep disturbance, more research on the effects of as-needed use of meditation apps is warranted.

### Limitations

Although this was the first study to report usage characteristics among a large sample of subscribers to a mobile app with and without self-reported sleep disturbance, there are some limitations to note. First, participants were mostly White, female, and highly educated, limiting generalizability across populations. Also, because participants had to have opened at least one email from Calm in the last 90 days, the sample was likely made up of highly engaged users of Calm. This is reflected in that, of those who opened the invitation, less than 15% chose to participate (3% of those who were sent emails to participate). Additionally, the large influx of traffic shortly following the release of email invitations temporarily overloaded the host server, and survey loading times were substantially slowed, such that survey completers were likely more committed to sharing their feedback compared to those who did not complete the entire survey. Second, as the sample had to have used a sleep component on the app in the last 90 days, we cannot assume the findings are representative of Calm users who do not use the app for sleep. However, even with this eligibility requirement, there was a portion of the sample who did not endorse sleep difficulties and those who did not use the app for sleep, such that we may be capturing at least some of the patterns associated with other uses of sleep-related content among good sleepers. Third, this was a cross-sectional survey, relying on participants’ retrospective self-reports. Future research that includes objective usage data in addition to self-reported usage would allow for a more nuanced understanding of these findings and broaden the range of research questions that could be explored. This may be particularly useful for addressing questions regarding if and how various types of sleep-related content within the app may be used differently, and the extent to which those with sleep difficulties prefer different types of sleep content than those without sleep difficulties. Additional research is needed to understand the extent to which these factors may drive or explain some findings presented here. Finally, the study was limited in scope because it did not address the efficacy of Calm’s sleep content. Given the potential for using mobile apps to deliver highly scalable, accessible interventions for sleep disturbance, future RCTs are warranted to determine the effectiveness of mindfulness meditation apps for sleep and to assess the usage and effects of different types of sleep content among those with sleep disturbance.

### Conclusions

We are the first to report how subscribers to the Calm meditation app use the app for sleep. Our data suggest that a high proportion of Calm subscribers experiencing sleep disturbance, including insomnia, use the app to improve their sleep. Poor sleepers and those with insomnia are specifically using Calm at night and on a regular basis, suggesting they are intentionally using the app for the self-management of sleep. We describe subscribers’ use of other pharmacological and nonpharmacological sleep aids, highlighting the extent to which sleep support is needed among Calm users experiencing sleep disturbance.

Our findings have important implications for future research and the development of content and interventions for meditation apps that aim to reduce sleep disturbance. There is a need for RCTs that can determine whether consumer-based meditation mobile apps are efficacious for improving sleep in those experiencing sleep disturbance, including those diagnosed with insomnia. Additional studies are necessary to determine the dose (ie, extent of app engagement) that is necessary for reducing sleep disturbance. Our findings also offer directions for designers of consumer-based apps that aim to develop content to help users fall asleep and return to sleep after waking in the night.

## Appendix

Multimedia Appendix 1 Sleep survey questions.

## Abbreviations

RCT: randomized controlled trial
SAB: Scientific Advisory Board
