# Supplementary material for: Use of the Consumer-Based Meditation App Calm for Sleep Disturbances: Cross-Sectional Survey Study
Source: JMIR Form Res. 2020 Nov 13;4(11):e19508. doi: 10.2196/19508 (PMC7695531; doi:10.2196/19508)
Supplement: Multimedia Appendix 1 [file formative_v4i11e19508_app1.docx]

How many times per **week** do you use Calm? < 1, 1, 2, 3, 4, 5, 6, 7+, I do not use Calm

How much do you feel about using Calm? Very much like - Very much dislike (5-point)

Why did you start using Calm?

- Improve overall health
- Reduce stress
- Reduce depression/anxiety
- Improve sleep
- Friend recommended the app
- Someone bought it for me
- Curious
- Other (please describe):

When you first started using Calm, did you have difficulty falling asleep or staying asleep?

- - Falling asleep
  - Staying asleep (i.e., staying asleep longer, waking up fewer times at night)
  - Waking too early
  - Getting a restful night sleep
  - None of the above (i.e., no difficulty sleeping)

What are some reasons why you think you have trouble sleeping?

- Racing thoughts or not being able to turn your mind off
- Stress or anxiety
- Nightmares
- Noise in the environment (e.g., snoring partner)
- Light in the environment (e.g., screens, electronics)
- Work or sleep schedule (e.g., working late at night, taking late-afternoon naps)
- Fluctuating hormones
- Physical pain or discomfort
- Medications that interfere with sleep
- Caffeine
- Other(s)
- I don't know

How many times per **week** do you use each component in order to help with your sleep? < 1, 1, 2, 3, 4, 5, 6, 7+, I do not use for sleep

- Sleep Stories
- Music/soundscapes
- Sleep meditations
- Meditaitons
- Breathe Bubble

How do you feel about using [component]? Very much like - Very much dislike (5-point)

Generally, when do you use Calm?

- - Within the 30 minutes after waking up in the morning
  - In the morning, but not within 30 minutes of waking up
  - In the afternoon
  - In the evening
  - At night, but not within 30 minutes of going to bed
  - Within the 30 minutes before laying down to go to bed at night
  - While laying down to go to bed at night (e.g., to fall asleep)
  - When I wake up during the night

Which best describes the way you use Calm at night?

- I try to use Calm at night on a regular basis
- I sometimes/occasionally use Calm at night
- I use Calm at night only when I need it (e.g., because of sleep difficulties that night)

When you use Calm at night because you need it, what do you usually need it for?

- - Falling asleep
  - Staying asleep (i.e., staying asleep longer, waking up fewer times at night)
  - Falling back to sleep after I wake up at night
  - Waking up too early
  - Getting a restful night sleep

At night, which component of Calm do you use the **most** often? [list components]

Overall, how much do you feel that Calm has helped improve your ability to... Very much improved – No noticeable improvement (3-point)

- Fall asleep
- Stay asleep (i.e., sleep longer, waking up fewer times at night)
- Not wake up too early
- Get a restful night sleep

Would you recommend using Calm to others with sleep difficulties? Y/N

[For components used at night] Do you think that using each of the components below has helped improve your sleep? Very much improved – No noticeable improvement (3-point)

[For components used at night] On nights that you *don’t* use these components of Calm, do you notice a difference in your sleep that night? Sleep is very much worse – No noticeable difference (3-point)

When I use Calm for sleep… Very much – Not noticeably (3-point)

- I wake up feeling refreshed
- I am better able to concentrate during the day
- I have more energy during the day
- I am more patient with my family and friends
- It is easier to make healthier food choices
- It is easier to engage in physical activity
- I experience a better mood

During the last 90 days: **Aside from Calm**, what other things have you used to help you sleep?

- Professional medical treatment (i.e., sleep specialist)
- Professional psychological treatment for sleep
- Prescription sleep medications
- Over-the-counter sleep medications (not melatonin)
- Melatonin
- Noise machine
- ASMR videos/audio
- Relaxation exercises/routines (e.g., progressive muscle relaxation)
- Yoga before bed
- Other relaxation apps (please describe)
- Other activities (please describe)

During the last 90 days: Are there other things that you have previously used to help you sleep, but you do not currently use or use less often as a result of using Calm? [see list above]

How old are you?

What is your race? [list]

Do you identify as Hispanic or Latino? Y/N

What is your gender? [list]

What is your annual household income?

What best describes your employment status? [list]

What is the highest level of education that you have completed? [list]

When you downloaded Calm, had you been diagnosed with any of the following chronic conditions?

- Anxiety disorder
- Posttraumatic Stress Disorder
- Depression
- High blood pressure (Hypertension)
- High cholesterol
- Diabetes
- Asthma
- Emphysema or COPD
- Other lung disease
- Heart disease
- Arthritis or other rheumatic disease
- Cancer
- Pain
- Insomnia
- Other chronic condition (please describe): __________
- None

[Of conditions endorsed]: Overall, do you think that using Calm helped improve any of your conditions below? Very much improved condition/symptoms – No noticeable difference (3-point)

Do you give your permission for the researchers to view individual information about your participation with the Calm app since you began your subscription (e.g., type of meditations used, length of meditation, etc.)? Your responses will not be linked to your Calm account information without your permission. Y/N
